# One-Year Follow-Up of Non-Healing Socket in Hodgkin’s Lymphoma Patient: Case Report and Literature Review on Management Strategies

**DOI:** 10.3390/diagnostics15101215

**Published:** 2025-05-12

**Authors:** Ahmed Ata Alfurhud

**Affiliations:** Oral and Maxillofacial Surgery and Diagnostic Sciences Department, College of Dentistry, Jouf University, King Khalid Road, Sakaka 72388, Saudi Arabia; dr.aalfurhud@jodent.org or aalfarhood@ju.edu.sa; Tel.: +966-501738443

**Keywords:** delayed healing, hodgkin disease, immunocompromised host, osteonecrosis, sodium hypochlorite, treatment outcome

## Abstract

**Background and Clinical Significance:** Sodium hypochlorite (NaOCl) is widely used in root canal treatment for its potent antiseptic and antibacterial effects. However, its cytotoxicity—particularly at higher concentrations and in patients with low immune status—has been associated with serious postoperative complications. This case report describes the risks associated with NaOCl exposure in a medically compromised patient and reviews the relevant literature on NaOCl-related injuries, offering insights into potential current management strategies. **Case Presentation:** This case report describes a challenging scenario of a 25-year-old male with a history of Hodgkin’s lymphoma who developed a non-healing bone in the lower right first molar (LR6) region after NaOCl exposure. Several months after undergoing root canal treatment and an extraction of the LR6, the patient presented with exposed necrotic bone in the region. The case’s complexity was heightened by the patient’s medical and dental history, which included chemotherapy and NaOCl exposure. Following a detailed clinical, radiographic examination and biopsy, the patient was diagnosed with bone necrosis due to NaOCl exposure. The treatment involved the extraction of the LR6, the debridement of the necrotic bone, and long-term follow-up with antimicrobial therapy. Despite efforts to manage the complication, the healing process was prolonged, potentially due to the patient’s immunocompromised state from chemotherapy. The patient’s condition remained unresolved after nearly a year, and ongoing management, including regular follow-up, was necessary to monitor healing and prevent further complications. This case highlights the challenges of treating dental complications in immunocompromised patients, particularly those with Hodgkin’s lymphoma, where delayed healing is a problem that might occur. **Conclusions**: Given the complexity of this case, different adjunctive treatment options, such as leukocyte–platelet-rich fibrin (L-PRF), pentoxifylline and tocopherol (PENTO), and hyperbaric oxygen therapy (HBOT), were discussed as potential treatments to help manage non-healing sockets in patients with similar conditions.

## 1. Introduction

Sodium hypochlorite (NaOCl) has been extensively utilized in dentistry for many years. Its use is critical for achieving successful root canal treatment, functioning as an effective antiseptic and antibacterial irrigating solution to significantly eliminate bacterial populations within the canal system [1]. The clinical technique centers on thorough cleaning and debridement, ensuring the removal of bacteria, necrotic tissue, and residual organic material, which is essential for optimizing treatment outcomes [1]. Nevertheless, concerns have been raised regarding the cytotoxicity of NaOCl, particularly at higher concentrations, with occasional reports of postoperative complications. Several studies have reported both temporary and permanent complications associated with the potential toxicity of NaOCl [1]. Serious complications have been documented in the literature, including unilateral facial swelling, persistent paresthesia in the affected region, and partial paralysis of the facial muscles [2]. Moreover, weakness of the buccal branch of the facial nerve has also been observed, leading to the loss of the nasolabial angle and a downward deviation of the right corner of the mouth [3].

The medical history of a patient is a critical factor in assessing the risk of complications. Medically compromised individuals are particularly at risk of experiencing severe adverse effects from NaOCl compared with healthy patients, as pre-existing systemic conditions may enhance its cytotoxicity, impair tissue repair mechanisms, and increase the likelihood of nerve damage and soft-tissue necrosis. Lymphoma is a diverse malignant condition of the lymphatic system, marked by the abnormal growth of lymphoid cells. Hodgkin’s lymphoma predominantly affects the lymph nodes, with more than 90% of cases occurring in these areas, while only 1–4% involve extranodal sites, usually presenting as a nodal disease with a preference for the neck and mediastinal nodes [4].

Diagnosing oral lymphomas can be difficult, as their symptoms often mimic those of other conditions like periodontal disease, osteomyelitis, and other cancers, which may result in delayed treatment and a poorer prognosis [4]. The current case presents significant diagnostic challenges due to the complexity of the patient’s medical history. Pre-existing conditions and underlying health factors complicate the identification of symptoms, making it difficult to distinguish between the primary disease, other potential comorbidities, and local complications, thereby complicating the diagnostic process.

## 2. Case Presentation

A 25-year-old male was referred to the Oral and Maxillofacial Surgery Department (OMFS) at the Royal London Dental Hospital (RLDH) with exposed necrotic bone in the lower right first molar (LR6) region. The patient had previously undergone root canal treatment on the LR6 in Romania several months ago. He later presented to a general dental practice in the United Kingdom with exposed bone in the LR6 area, reporting no pain but experiencing an unpleasant taste. Concerned about the exposed necrotic bone in the LR6 socket and the patient’s complex medical history, the general dentist referred him to the OMFS Department for further evaluation and management. The patient’s medical history included a diagnosis of Hodgkin’s lymphoma (stage 2), for which he underwent a six-month course of chemotherapy, completed in September 2020. Additionally, he experienced an endodontic irrigation material-related incident in the LR6 region, which may have contributed to the development of complications in the area.

### 2.1. Clinical Examination

**Figure 1:**
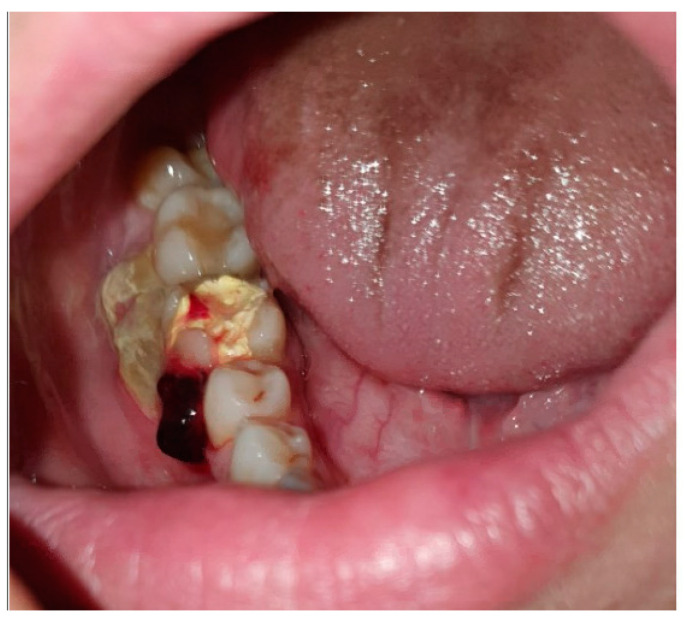
A preoperative photograph of the LR6.

Intraorally, a preoperative photograph (Figure 1), provided by the general dentist who initially examined the patient, revealed a temporary restoration on the LR6. Additionally, there was an area of exposed, non-healing bone on the buccal aspect, extending from the mesial surface of the LR6 to the distal surface of the lower right second molar. A relative blood clot was observed on the mesial surface of the LR6, with no identifiable cause.

**Figure 2:**
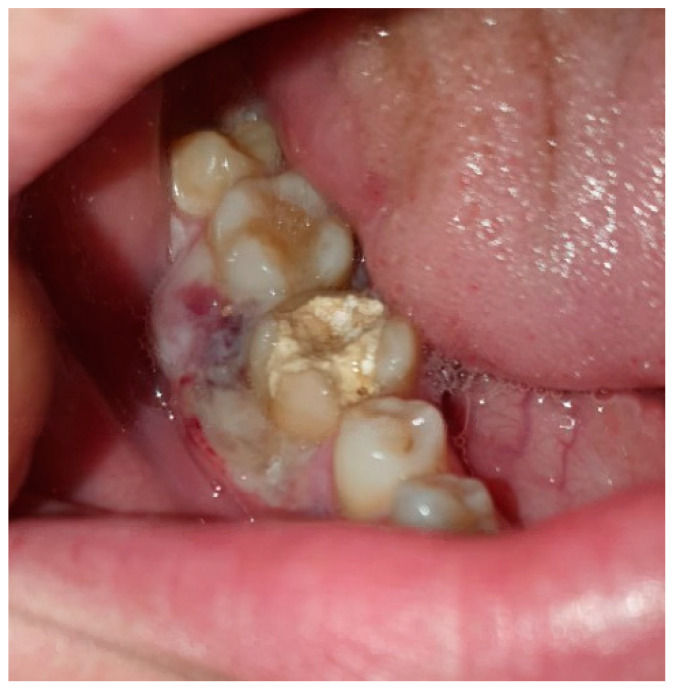
A preoperative photograph of the LR6 during the first follow-up visit.

A few weeks later, the patient was evaluated by a specialist at the OMFS Department at the RLDS. A clinical examination revealed non-healing exposed necrotic bone in the LR6 region, as illustrated in Figure 2. Additionally, signs of infection were evident, including pain, purulent discharge at the LR6 site, and mild extraoral swelling on the lower right side of the face.

### 2.2. Differential Diagnosis

The clinical findings were suggestive of a differential diagnosis, as follows: the most likely diagnosis in this case was bone necrosis due to a sodium hypochlorite incident, as the patient had a history of an endodontic irrigation-related incident in the LR6 region. Sodium hypochlorite is known for its cytotoxic effects, and accidental extrusion beyond the root canal system can lead to severe tissue damage, necrosis, and delayed healing, which aligns with the patient’s clinical presentation of exposed necrotic bone.

However, other differential diagnoses could not be excluded at this stage. Chronic osteomyelitis remains a possibility, as persistent infection in the LR6 area may lead to necrotic bone, purulent discharge, and localized swelling. Additionally, primary or secondary malignancy, such as lymphoma or metastatic carcinoma, should be considered. Lastly, traumatic bone sequestration could also be a contributing factor, particularly if prior dental procedures caused localized trauma, leading to devitalized bone fragments and subsequent exposure.

### 2.3. Management and Intervention

The patient was advised on improving oral hygiene practices to help prevent the progression of bone necrosis, which included proper brushing techniques, antimicrobial mouth rinses, and regular dental check-ups to maintain oral health and minimize the risk of further complications. Given the clinical evidence of infection, a 14-day course of oral 500 mg of amoxicillin, three times a day, was prescribed to control the infection and reduce the bacterial load in the affected area. As the LR6 was deemed non-restorable due to its fragility after the root canal treatment and the presence of a large, mobile, fractured fragment, a simple extraction was performed under local anesthesia to eliminate the source of infection and promote healing, with careful attention to minimizing trauma. A biopsy of the necrotic bone and surrounding mucosa was undertaken to exclude malignancy. Additionally, an ostectomy of the buccal plate of bone (buccal cortex) was performed, followed by curettage of the necrotic bone. The patient was placed on a long-term follow-up protocol to monitor their healing, assess for any recurrence of necrotic bone, and ensure no signs of persistent infection or malignancy, with regular clinical and radiographic evaluations scheduled to track progress and intervene if necessary.

### 2.4. Microscopic Description

Gross examination of the biopsy specimen revealed both hard and soft tissues, with irregular, discolored, and friable bone and mucosa. Histopathological analysis showed acellular necrotic bone, characterized by empty lacunae and loss of osteocytes, surrounded by bacterial aggregates and a mixed inflammatory infiltrate. The bacteria were interspersed with neutrophilic infiltrates and fragments of granulation tissue, with collections of multinucleated osteoclast-type giant cells also observed. The adjacent mucosal biopsy consisted of tissue fragments covered by hyper-parakeratinized epithelium, exhibiting variable hyperplasia, fibrin deposits, and chronic inflammatory cell infiltration. The epithelium demonstrated neutrophilic trafficking and spongiosis, with no significant cytological atypia. Dense infiltrates of neutrophils, lymphoplasmacytic cells, and histiocytes were present, along with scattered hemosiderin deposits. No evidence of epithelial dysplasia or malignancy was identified. The findings are consistent with chemically induced necrosis due to sodium hypochlorite exposure.

### 2.5. Follow-Up and Outcomes

**Figure 3:**
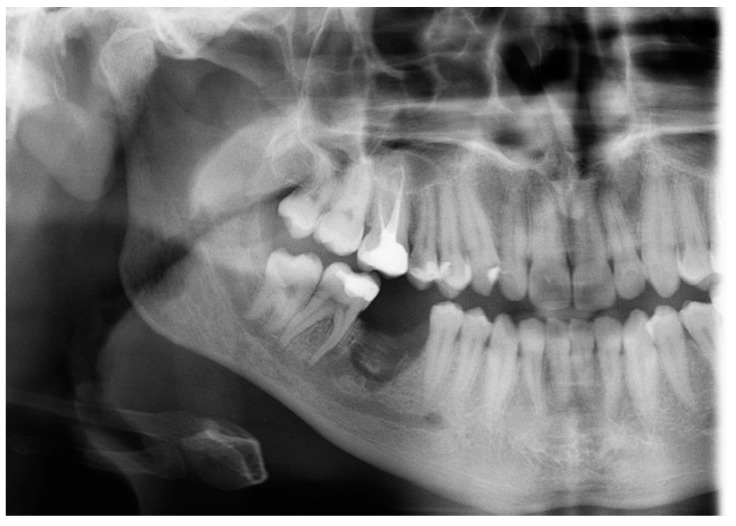
A postoperative radiograph of the LR6 during the follow-up visit.

The initial radiographic assessment was conducted a few weeks after the surgery. As shown in Figure 3, the orthopantomograph (OPG) revealed a non-healing bony defect within the extraction socket. This defect extended inferiorly, encroaching upon the inferior alveolar canal, which may suggest the potential involvement of the nerve. Notably, there was an increase in the bony density adjacent to the socket, consistent with sclerosing osteitis. These findings necessitate further radiographic evaluation and monitoring to assess the progression of healing and any potential complications.

**Figure 4:**
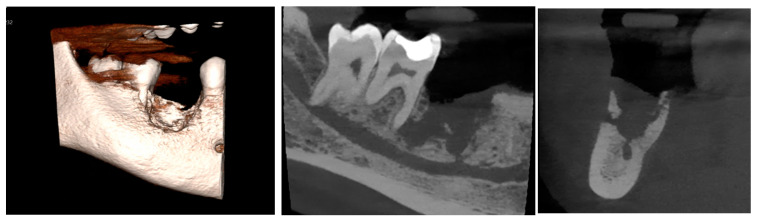
A postoperative CBCT of the LR6 during the follow-up visit.

A cone-beam computed tomography (CBCT) scan of the posterior mandible revealed a bony sequestrum in the region of the missing LR6 socket, as shown in Figure 4. There was a subtle periosteal bone reaction on both the buccal and lingual aspects of the socket, along with a marked increase in the adjacent bony density. The bony defect extended inferiorly to involve the inferior alveolar canal, with a small dehiscence of its superior cortex. Additionally, the defect extended mesially to the root of the LR7, with apical periodontal ligament space widening at both LR7 apices. The inferior alveolar canal was in contact with the buccal aspect of the radiolucency associated with the distal apex of the LR7, and there was partial loss of the canal’s cortical outline in this region.

Although the definitive diagnosis, confirmed by the histopathological examination, was necrosis due to sodium hypochlorite exposure, the patient required ongoing regular follow-up to monitor the healing process. Given the patient’s complex medical history, including prior chemotherapy, there was an increased risk of delayed healing, secondary infection, and potential complications, such as osteomyelitis. Regular clinical and radiographic assessments are essential to evaluate bone regeneration, soft-tissue healing, and any signs of persistent inflammation or further necrosis.

The primary objective in this case was to achieve primary closure to minimize the risk of infection at the surgical site. However, this was not possible due to the compromised healing process, likely resulting from the patient’s history of chemotherapy. Chemotherapy can impair wound healing by affecting cellular regeneration, reducing vascular supply, and compromising immune response, increasing the risk of delayed epithelialization and secondary infection. As a result, the open wound remained vulnerable to bacterial colonization and further necrosis.

**Figure 5:**
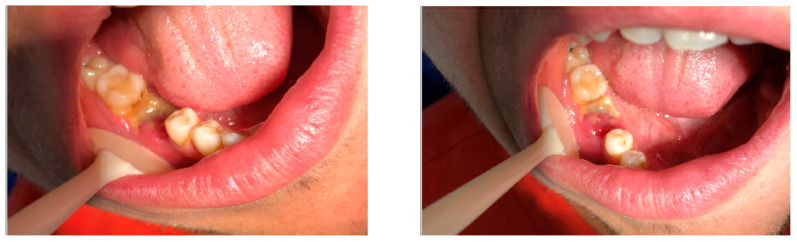
A postoperative clinical photograph of the LR6 area, taken one year after the surgery, showing the healing status of the site.

As can be seen in Figure 5, the healing process in this case was prolonged and complex. During follow-up, the surgical site showed minimal progress, with no complete healing observed. Despite nearly a year of monitoring, primary closure was not achieved, likely due to impaired tissue regeneration and delayed healing associated with the patient’s medical history. This necessitated continued follow-up and potential adjunctive interventions to support tissue repair and minimize the risk of further complications.

The clinical findings, as shown in Figure 5, were recorded during the patient’s last attended appointment, one year after the surgery. Unfortunately, the patient did not return for the remaining follow-up appointments for unknown reasons, making it challenging to assess the long-term healing progress and any potential complications.

### 2.6. Review of the Literature

Several case reports identified in the literature review, summarized in Table 1, describe a wide range of complications associated with NaOCl exposure. Most of these complications are typically uncomplicated and resolve within a few days to weeks; however, some complications may persist for a prolonged period or even become permanent. Accidental exposure to NaOCl during endodontic procedures typically results in mild to moderate complications, including pain and swelling, which generally resolve within a period ranging from several days to weeks. However, some severe symptoms, such as skin discoloration, chemical burns, and mucosal or bone necrosis, may require a prolonged healing period, with full recovery potentially taking up to months. Accidental NaOCl exposure can result in severe, long-lasting, or permanent complications, such as sensory impairment, paresthesia, facial nerve dysfunction and facial muscle weakness potentially persisting for years or even becoming permanent. Therefore, most NaOCl-related injuries are self-limiting, with the majority healing completely within a few weeks. However, in severe cases involving extensive nerve tissue damage, the healing period may extend for a long time. A summary of the included case reports is provided in Table 1.

## 3. Discussion

Identifying the risk factors that predispose individuals to prolonged bone necrosis following NaOCl exposure is essential for both prevention and effective management. The presented case led to bone necrosis and a non-healing extraction socket, with the complication persisting for a much longer period than expected. This extended healing time exceeded the typical duration described in the literature, indicating that there may be additional factors influencing wound healing and tissue repair, which likely contributed to the prolonged nature of the complication. Individuals with impaired immune function or those undergoing immunosuppressive treatment are at a significantly higher risk of developing severe complications following NaOCl injuries [17]. In immunocompromised patients, such as those with Hodgkin’s lymphoma, a usually localized area of tissue necrosis may progress more rapidly or show delayed healing.

The clinical outcome of a NaOCl accident is largely influenced by patient-related factors, such as medical history. This factor determines whether the injury remains a self-limiting or transient event in otherwise healthy patients or progresses into more severe complications, such as persistent osteonecrosis, as demonstrated in the presented case.

In patients with a healthy immune status, NaOCl injuries typically present with acute pain and swelling, which generally resolve with conservative management [5,7,9,10,11,13,14,15,16]. In contrast, the presence of a high-risk factor, particularly immunocompromised states, such as in patients with Hodgkin’s lymphoma, can dramatically alter the clinical trajectory. In such individuals, a compromised inflammatory response and impaired ability to contain secondary infection may lead to more extensive soft-tissue destruction and involvement of the alveolar bone [17], even when procedural guidelines are followed and predisposing conditions and risk factors are carefully considered [18]. These complex cases often require surgical intervention, such as debridement of the necrotic bone, along with prolonged wound care and monitoring to aid healing, as was performed in the presented case. However, despite these efforts, the desired outcomes were not achieved.

Notably, no clinical cases documenting NaOCl injuries in patients with Hodgkin’s lymphoma have been identified in the existing literature. Consequently, there is no specific management protocol designed for this patient group. This poses a clinical challenge, as extended monitoring in such an individual may increase the risk of secondary infection over time, while early surgical intervention, such as debridement of the necrotic bone, could potentially worsen the condition by causing further bone damage.

Therefore, as the conventional management approach failed, this patient should be managed according to the most up-to-date treatment strategies, as suggested in situations similar to those involving Hodgkin’s lymphoma patients, where individuals exhibit a low immune status.

One of the recently established therapies for managing non-healing sockets, particularly in the context of medication-related osteonecrosis of the jaw (MRONJ) [19], is leukocyte–platelet-rich fibrin (L-PRF). This treatment has demonstrated promising outcomes in addressing non-healing sockets in non-malignant conditions, such as osteoporosis, and oncology patients, offering a potential solution for enhancing healing and improving clinical outcomes.

A retrospective observational study [19] was conducted to evaluate the effectiveness of L-PRF as an adjunct to surgical intervention in the treatment of patients at risk of MRONJ development. The study included 22 participants, who were allocated into two groups: Group A, which received surgery alone, and Group B, which received both surgery and L-PRF. Treatment success was determined based on complete soft-tissue healing and the absence of infection, inflammation, fistula formation, or exposed bone. The results demonstrated that 100% of patients in Group B achieved complete healing, in contrast with 54.5% of patients in Group A. The study concluded that L-PRF is a promising adjunct to the surgical management of MRONJ, providing favorable treatment outcomes and enhanced healing potential. A similar study [20] found that none of the patients in the L-PRF group developed established MRONJ, while five high-risk patients in the control group presented with MRONJ during follow-up. The study concluded that L-PRF may be effective in preventing MRONJ following dental extractions and also introduced a management protocol for these individuals.

Another group of bone necrosis and non-healing sockets is observed in osteoradionecrosis of the jaws (ORNJ), a severe and complex complication that arises following head and neck radiation therapy. In the management of ORNJ, one promising therapeutic option is the combination of pentoxifylline and tocopherol (PENTO). A systematic review [21] was conducted to assess the efficacy of PENTO in the treatment of ORNJ. The review revealed that PENTO treatment resulted in complete mucosal coverage with no exposed bone in patients, with success rates ranging from 16.6% to 100%, depending on the study. Additionally, clinical improvement or disease stabilization was reported in 7.6% to 66.6% of patients, while disease progression occurred in 7.6% to 32% of cases across five studies. The findings of the review suggest that PENTO may effectively achieve complete disease control in a significant proportion of patients.

Another treatment approach for ORNJ involves addressing radiation tissue injury. Hyperbaric oxygen therapy (HBOT) has been proposed as a potential treatment for such complications, owing to its ability to enhance blood flow to irradiated tissues. A Cochrane systematic review [22] was conducted to evaluate the effectiveness and risks of HBOT in treating or preventing late radiation tissue injury. The review found that HBOT is associated with improved outcomes in managing osteonecrosis. Additionally, it appears to reduce the risk of osteoradionecrosis following tooth extraction, indicating its potential benefit as an adjunctive therapy for patients with radiation-induced complications.

These adjunctive management strategies have been utilized in the treatment of various medical conditions, such as MRONJ and ORNJ, which share similar underlying pathophysiologies of NaOCl injury. Additionally, these conditions exhibit notable parallels to the present case, particularly in the manifestation of bone necrosis and delayed wound healing in immunocompromised patients.

The pathophysiology of NaOCl injury is predominantly attributed to its potent cytotoxic effects on living tissues. When NaOCl is accidentally extruded beyond the confines of the root canal during endodontic procedures, it comes into direct contact with surrounding vital tissues, leading to a cascade of harmful biological responses. NaOCl induces hemolysis by disrupting the integrity of red blood cells and releasing their intracellular contents into the surrounding tissues, thereby contributing to the disruption of the local tissue environment [14]. Additionally, NaOCl causes the ulceration of the affected tissue, leading to a compromised mucosal barrier [14]. The resultant disruption of the mucosal integrity creates a vulnerable site where microorganisms can more easily penetrate the exposed underlying bone, potentially leading to secondary infection. One of the key mechanisms of injury is the inhibition of neutrophil migration, which diminishes the immune response and compromises the body’s ability to combat infection and initiate the healing process [14]. Furthermore, NaOCl inflicts damage to endothelial cells, disrupting vascular integrity, and fibroblast cells, hindering tissue repair and collagen synthesis [14].

As outlined in the 2022 update of the Position Paper by the American Association of Oral and Maxillofacial Surgeons on MRONJ, the pathophysiology of MRONJ is supported by several evidence-based theories. The pathophysiology of MRONJ is multifactorial, involving several interrelated mechanisms. One major factor is the inhibition of bone remodeling due to antiresorptive medications, which directly affect osteoclast formation, differentiation, and function, particularly following bone injury. Additionally, pre-existing inflammation or infection contributes significantly, with elevated levels of pro-inflammatory cytokines—especially at MRONJ lesion sites—supporting the pivotal role of inflammation in disease progression. Inhibition of angiogenesis also plays a key role, characterized by osteocyte death, secondary to reduced blood flow, and a notable decrease in the microvessel density during the early stages of bone healing. Immune dysfunction, such as that caused by chemotherapy, further exacerbates the condition by altering the number and distribution of T-cell populations. Lastly, genetic predisposition has been implicated, with specific single-nucleotide polymorphisms identified as being associated with an increased risk of developing MRONJ [23].

The pathogenesis of ORNJ is primarily driven by the progressive changes induced by radiation therapy. A key mechanism involves the development of hypovascularity, hypocellularity [24], and hypoxia [21] in the irradiated bone. These changes compromise the tissue’s ability to regenerate and resist injury, initiating a chronic fibro-atrophic process characterized by fibrosis, reduced tissue elasticity, and impaired wound healing [24]. This leads to the bone and surrounding soft tissues becoming fragile and highly susceptible to necrosis, particularly following trauma, such as tooth extractions [24]. While microorganisms do play a role in exacerbating the condition, their contribution is relatively minor compared with the primary causes of hypovascularity and hypocellularity. Infection can worsen the clinical manifestation of ORNJ [25].

Therefore, the management strategies for MRONJ and ORNJ may be considered applicable in the present case, given the shared elements in their underlying pathophysiological mechanisms. L-PRF [26,27], one of the proposed strategies in the present case, is an autologous platelet concentrate, and regenerative biomaterial composed of a dense fibrin matrix containing high concentrations of platelets and leukocytes. It demonstrates considerable potential in promoting tissue repair through the gradual release of key growth factors, such as platelet-derived growth factor (PDGF), transforming growth factor (TGF), vascular endothelial growth factor (VEGF), and epithelial growth factor (EGF). These molecules collectively enhance angiogenesis, bone regeneration, and soft-tissue healing while also supporting hemostasis and tissue maturation without eliciting an inflammatory response. The fibrin matrix of L-PRF, with its tetra-molecular structure, serves as a biodegradable scaffold containing cytokines and stem cells, facilitating microvascularization and directing epithelial cell migration during the healing process.

Pentoxifylline, a methylxanthine derivative, exerts its antifibrotic effect through multiple mechanisms, including the inhibition of tumor necrosis factor-alpha (TNF-α), promotion of vasodilation, enhancement of erythrocyte deformability, and suppression of fibroblast proliferation [21]. Additionally, it increases the activity of collagenase enzymes, thereby facilitating extracellular matrix degradation [21]. Tocopherol (vitamin E), a potent antioxidant, complements these effects by inhibiting oxidative stress and downregulating the expression of procollagen genes, which contributes to the reduction in fibrotic tissue formation [21]. Together, pentoxifylline and tocopherol (PENTO) demonstrate a synergistic interaction, enhancing their overall antifibrotic efficacy.

HBOT has been proposed as a modality to enhance tissue quality, facilitate wound healing, and prevent the breakdown of irradiated tissues. It is defined as the medical use of 100% oxygen delivered at pressures exceeding 1 atmosphere absolute. The procedure involves placing the patient within a sealed hyperbaric chamber, where the ambient pressure is elevated while the patient breathes pure oxygen [22]. This results in a substantial increase in the partial pressure of oxygen in the lungs, bloodstream, and tissues. One of the key therapeutic outcomes of HBOT is the stimulation of angiogenesis, thereby promoting neovascularization within previously irradiated and hypoxic tissues [22].

Although no studies to date have directly investigated the use of these adjunctive management strategies in the context of NaOCl injuries, their therapeutic objectives—particularly the promotion of mucosal coverage over exposed bone—suggest potential applicability in such cases, especially when the patient is immunocompromised due to underlying medical conditions or ongoing treatment, as demonstrated in the presented case.

The dental management of patients with compromised immune systems presents unique challenges. These challenges arise not from the procedures themselves but from the complexities of managing complications in these patients. The current case emphasizes the necessity of careful consideration and expertise in treating such patients. Inexperienced clinicians should exercise caution when managing these cases, as complications could lead to long-term issues without effective management solutions. Hodgkin’s lymphoma, in particular, requires specialized attention due to the significant immune compromise associated with the condition. Therefore, a comprehensive and well-coordinated approach is crucial for the successful management of dental complications in this patient group. In this context, it may be advisable to consider the adjunctive management strategies discussed, as they could potentially aid in resolving the complications and promoting optimal healing. However, further well-designed research is required to evaluate the effectiveness of the proposed adjunctive management approaches and determine their suitability for use in such cases as Hodgkin’s Lymphoma.

## 4. Conclusions

This case report highlights a challenging clinical scenario commonly encountered in dental practice. While many dental procedures are straightforward, the medical history, particularly in immunocompromised patients, can present significant challenges. It is recommended that such patients be referred to specialists rather than managed by less-experienced clinicians in order to avoid potential long-term complications. The management approaches employed in other conditions, such as MRONJ, may offer valuable insights for managing similar cases to the presented case. Furthermore, a standardized management protocol should be established for handling such cases to ensure optimal patient outcomes.

## Figures and Tables

**Table 1:** A summary of the case reports discussed in this paper.

Reference	Signs and Symptoms	Duration	Managements	Outcomes
[2]	Pain and swelling, paresthesia in the infraorbital nerve area and weakness in the buccal branch of the facial nerve, leading to loss of upper lip and cheek function.	Pain and swelling lasted for about 1 week. Paresthesia persisted for 3 years with no signs of improvement in the facial nerve weakness.	Local anesthesia, cold compress, antibiotics, and analgesics. The patient was later referred to a neurologist for further assessment of the persistent sensory and motor deficits. The patient underwent prosthetic rehabilitation to replace the upper front teeth.	The patient experienced permanent facial nerve weakness and reduced sensitivity in the infraorbital region. No significant improvement occurred even after 3 years, despite daily attempts to train the mimic musculature. Permanent paresthesia and weakness of the mimic musculature, causing difficulty with facial movements like smiling and uncontrolled salivation.
[3]	Pain and swelling, weakness of the buccal branch of the facial nerve, leading to loss of upper lip and cheek function, with the mouth corner being pulled down by unopposed lower lip muscles. Infra-orbital ecchymosis and altered sensation in the right infra-orbital nerve area.	One month later: Swelling had almost resolved, mouth opening was improved, and the patient was pain-free. However, there was no improvement in facial nerve weakness after one month.	The patient was treated with intravenous dexamethasone (8 mg, three times a day) and co-amoxiclav (1.2 g, three times a day), along with regular analgesia.	Outcome after one month: The patient was free from pain, with significant improvement in mouth opening. However, the weakness of the facial nerve persisted, and there was minimal improvement in infra-orbital nerve paresthesia.
[5]	Burning sensation around the rubber dam during sodium hypochlorite irrigation. A rash developed around the patient’s chin, which later formed scabs.	Burning sensation ceased after 3 days, and the scab began to fall off after 7 days. The skin discoloration from the burn disappeared after 3 months, and the patient fully recovered.	The patient was treated with topical Hamamelis virginiana extract (Hametan) twice a day for 2 weeks.	The patient fully recovered with no long-term effects. The burning sensation and skin discoloration were alleviated, and there were no further complications after 3 months.
[6]	Pain and massive edema on the right cheek and upper lip. The swelling extended to the right orbit, and the patient experienced right eye pain, blurred vision, and corneal discoloration. A necrotic area appeared on the upper lip mucosa.	The symptoms developed rapidly within minutes to hours, with the swelling extending over two weeks.	The patient received intravenous hydrocortisone and penicillin G for swelling and pain control. Surgical debridement was performed to excise the necrotic mucosa and clean the affected area. The area was irrigated daily with saline to remove necrotic tissue, and intravenous antibiotics were administered to treat the infection.	After two weeks, the wound healed, but the patient experienced long-term scarring, dimples, and right infraorbital nerve anesthesia.
[7]	The patient reported burning, stinging, and sharp pain during the injection of 1% sodium hypochlorite. Edema, difficulty swallowing, redness on the cheek, and loss of sensation were noted in the mental nerve area.	The patient underwent regular debridement of necrotic tissue, and re-epithelialization was observed by the third month. All symptoms resolved by the sixth month.	The patient was administered pheniramine maleate, dexamethasone, and intraoral antibiotics (1000 mg of amoxicillin, twice a day, for two weeks), along with alpha-lipoic acid (300 mg, daily, for one month). Regular clinical follow-up was performed, and necrotic tissue was debrided every 3 days for 4 weeks. The patient was advised to avoid smoking and consuming hot food and alcohol.	Re-epithelialization of necrotic tissue was observed after one month. The paresthesia of the mental nerve decreased after three months, and all symptoms completely disappeared by the sixth month.
[8]	Severe chemical burn on the right infraorbital area and partial necrosis of the hard palate. Erythematous, tender skin on the right cheek, numbness, and blackish discharge from the nose.	Mucosal damage healed completely after six weeks, but facial scar discoloration remained.	The patient was treated with creams and ointments for chemical burns. The symptoms were managed conservatively without surgical intervention. The patient was monitored for two weeks and did not require further surgical treatment.	The facial scar remained, but the mucosal damage healed nearly completely. The patient recovered without significant long-term complications, though facial discoloration remained.
[9]	Case 1: Gross left facial swelling extending from the mandible to the zygomatic arch with diffuse subcutaneous ecchymosis, mouth-opening limited to 20 mm, and intra-oral necrosis, ulceration, and ecchymosis. Case 2: Severe pain and swelling in the left cheek, extending from the mandible to the left eye, with no intra-oral tissue damage.	The swelling and pain reduced significantly within a few days. In Case 1, mouth opening and facial swelling improved by the 7th day, and by the 3rd week, and the face and mouth were normal. In Case 2, the symptoms improved significantly within 4 weeks.	Case 1: The oral cavity was irrigated with normal saline, and ice packs were applied for 1 day, followed by warm saline rinses. Prednisone was prescribed to control inflammation, and co-amoxiclav and analgesics were given. Regular monitoring was undertaken over the next few days. Case 2: Cold and warm compresses, saline rinses, acetaminophen-based narcotic analgesics, prophylactic antibiotics, and corticosteroids were prescribed.	Case 1: Complete resolution of symptoms, normal mouth opening, and absence of pain by 3 weeks. Case 2: All symptoms resolved satisfactorily after 4 weeks. The root canal treatment was successfully completed with alternative irrigants (hydrogen peroxide and chlorhexidine gluconate).
[10]	Severe burning pain, swelling of the surrounding tissue, and redness observed at the tissues beyond the root apex. Profuse bleeding, severe tissue necrosis, and potential nerve involvement.	Symptoms improved appropriately, but full resolution may take several weeks, and nerve recovery could take several months.	Immediate irrigation with normal saline to flush out the NaOCl, followed by ice packs to reduce swelling. Steroids like prednisone were administered to control inflammation. Antibiotics to prevent secondary infections were prescribed, along with warm compresses to stimulate healing and improve circulation after the initial 24 h period.	The patient showed significant improvement over several weeks, with the swelling and ecchymosis reducing. In some cases, tissue necrosis was resolved after several weeks with non-surgical interventions, but facial nerve damage (e.g., numbness or weakness) could persist for a few months.
[11]	Sudden onset of pain in the left hemimandible and numbness in the left lower lip. Persistent pain in the left lower molar region and the left lower lip remained numb after the initial procedure.	Pain and numbness persisted for 15 days. After the gutta-percha point was removed surgically, pain subsided within 15 days, but numbness in the lower lip persisted for about a month before resolving completely.	A surgical procedure was performed to remove the gutta-percha point that had overfilled the root canal and entered the mandibular canal. The patient underwent an outpatient surgical procedure to remove the gutta-percha point, with the lesion being addressed by raising a mucoperiosteal flap for ostectomy and careful curettage.	The pain was resolved after the surgical removal of the gutta-percha, although the paresthesia (numbness) persisted for a while. The numbness in the lower lip was fully recovered within a month after the surgery.
[12]	Severe pain at the injection site, which began immediately after the sodium hypochlorite was injected instead of the anesthetic solution. Rapid development of local edema and tissue necrosis, labial ptosis (drooping), paresthesia in the upper lip, and visual blurring.	The severe pain and edema appeared immediately. The visual discomfort resolved after 8 days. The mucosal injury healed with scarring after 60 days. The labial ptosis resolved after 3 months, but the lip paresthesia persisted for 3 years.	The patient was given Arcoxia (anti-inflammatory), amoxicillin (antibiotic), and dexamethasone with B-complex vitamins for inflammation control. The patient received a combination of antibiotics and anti-inflammatory medications to manage the necrosis and prevent infection. Regular follow-up ensured proper tissue healing and symptom management.	The edema and necrosis resolved with scarring. Lip ptosis improved within 3 months, but paresthesia in the lip persisted for 3 years despite treatment.
[13]	Severe pain upon injection, which was followed by tissue necrosis in the palatal mucosa. Swelling and discoloration (purple) around the necrotic area, with the center being yellow-white. The area was painless during palpation.	Pain persisted for 2 days after the injection, after which it subsided. Swelling and tissue necrosis were visible for the next 15 days.	The patient was monitored conservatively as the mucosa showed signs of healing. No surgical intervention was needed since the tissue was healing naturally. Regular monitoring was carried out for 30 days, ensuring that no further complications arose.	Complete healing without scarring was achieved within 30 days. No long-term complications were reported, and the mucosal tissue healed effectively.
[14]	Severe pain occurred immediately after the accidental injection of NaOCl instead of the intended anesthetic solution. Swelling of the gingiva and surrounding tissues, followed by the formation of necrotic tissue and bone sequestration.	Pain persisted for 3 days after the injection. Swelling and tissue necrosis were present for the following 2 months.	The patient was treated with anti-inflammatory medication (diclofenac) and antibiotics (amoxicillin) to manage pain, inflammation, and prevent infection. After 2 months, surgical coverage with a laterally positioned flap was performed to address the necrotic tissue and bone sequestration.	The area affected by chemical necrosis healed after 3 weeks, with no further tissue damage or complications. The root canal treatment of the adjacent tooth was completed 30 days after the surgical procedure.
[15]	Severe pain upon injection, swelling, and hyperemia in the right half of the face, along with black necrotic areas on the right buccal mucosa. No facial paralysis, but the patient exhibited significant tissue damage and edema in the affected area.	Severe swelling, pain, and necrosis developed within 4–5 h of the accidental injection. After 24 h of treatment, the patient’s symptoms regressed. Full recovery occurred within 4 weeks, with no residual effects.	The patient was hospitalized and treated with IV antibiotics (ceftriaxone and metronidazole), anti-inflammatory medication (dexketoprofen), and analgesics (paracetamol). Cold compresses and bed rest were recommended. The patient was monitored for 4 weeks, after which they showed complete recovery with no complications.	The patient recovered fully without any long-term effects, and the necrotic tissue healed completely within a month. There were no additional issues reported after the treatment.
[16]	Report A: Swelling and ecchymosis on the right side of the face, particularly around the mouth and periorbital region. Ulcerative lesions on the internal lip mucosa. Report B: Mild edema and ecchymosis on the right commissure lip with no mucosal lesions. Report C: Swelling and ecchymosis on the left side of the face, particularly near the mouth and periorbital region. Report D: Severe pain and bleeding from the root canal, followed by mild edema and swelling on the face.	Report A: Swelling and bruising peaked within 24 h, which improved significantly after 3–5 days. Report B: Swelling and ecchymosis persisted for 5 days but resolved with minimal symptoms remaining. Report C: Symptoms like swelling and ecchymosis reduced after 5 days. Report D: Swelling persisted for several days, but pain and symptoms resolved within 6 days.	Report A: Cold compress, antibiotic (amoxicillin with clavulanic acid), pain control (paracetamol and ibuprofen), and corticosteroids (prednisone). Report B: Saline solution flush, cold compress, and prescribed medications (ciprofloxacin, paracetamol, ibuprofen, and betamethasone). Report C: Saline flush, cold compress, and medications (amoxicillin, prednisone, and ibuprofen). Report D: Saline flush, cold compress, and prescribed medications (amoxicillin, prednisone, and ibuprofen).	Report A: Significant improvement in 3–5 days, with the continuation of root canal treatment using chlorhexidine. Report B: Symptoms reduced significantly after 5 days; root canal treatment continued with chlorhexidine. Report C: Recovery within 5 days, root canal treatment continued with a side-vented needle after CBCT revealed apical fenestration. Report D: Significant reduction in symptoms after 6 days, treatment completed with no legal actions taken.

## Data Availability

The data concerning this article may be requested from the corresponding author for reasonable purposes.

## List of Abbreviations

LR6	Lower right first molar
NaOCl	Sodium hypochlorite
L-PRF	Leukocyte–platelet-rich fibrin
PENTO	Pentoxifylline and tocopherol
HBOT	Hyperbaric oxygen therapy
OMFS	Oral and maxillofacial surgery department
RLDH	Royal London Dental Hospital
OPG	Orthopantomograph
CBCT	Cone-beam computed tomography
MRONJ	Medication-related osteonecrosis of the jaw
ORNJ	Osteoradionecrosis of the jaws
